# Mobilization of Readily Exchangeable Organic P – A Potential Driver of Enhanced P Acquisition Efficiency in Upland Rice

**DOI:** 10.1007/s42729-025-02467-z

**Published:** 2025-05-19

**Authors:** Eva Mundschenk, Rainer Remus, Matthias Wissuwa, Christiana Staudinger, Uxue Otxandorena-Ieregi, Eva Oburger, Maire Holz

**Affiliations:** 1 Group of Root Soil Interaction, Leibniz Centre for Agricultural Landscape Research (ZALF) E.V, Müncheberg, Germany; 2 Department of Crop and Animal Sciences, Plant Nutrition, Humboldt University of Berlin, Unter Den Linden 6, 10099 Berlin, Germany; 3 Japan International Research Center for Agricultural Sciences (JIRCAS), 1-1 Ohwashi, Tsukuba, Ibaraki 16 305-8686 Japan; 4 Department of Forest and Soil Science, Institute of Soil Research, University of Natural Resources and Life Sciences, 3430 Tulln an Der Donau, Austria; 5 Department of Crop Sciences, Institute of Soil Research, University of Natural Resources and Life Sciences, 3430 Tulln an Der Donau, Austria; 6 Present Address: PhenoRob Cluster & Institute of Crop Science and Resource Conservation, Bonn University, Bonn, Germany

**Keywords:** Rhizosphere soil, Hedley fractionation, Andosol, Upland rice, Radioisotope tracer

## Abstract

High phosphorus (P) fixation in soils is a major constraint on crop production worldwide. To address this challenge, we investigated plant-induced changes in soil P pools, aiming to identify superior P uptake strategies by examining whether different upland rice genotypes access various P sources in the rhizosphere. Two genotypes (DJ123 and Nerica4) with varying P acquisition efficiencies (PAEs) were grown in an Andosol under low- and high-P fertilization. Fertilizer-P was labeled with 33P, and plants were harvested 34 days after emergence. Hedley fractionation was conducted on initial soil, as well as on bulk and rhizosphere soils after harvest, to analyze changes in fertilizer/native soil and inorganic/organic P in different fractions. Fertilizer-P entered all Hedley fractions, with the largest share being found in the moderately labile (NaOH-P, + 72%) and stable (H2SO4-P, + 19.8%) P fractions under both P treatments. The plant presence resulted in a decrease in fertilizer-P in the most labile P fraction (resin-P), whereas native soil-P and organic P increased in the other labile P fraction (NaHCO3-P). Moreover, a sharp decline in organic NaOH-P fraction in the rhizosphere, along with an increase in inorganic NaOH-P under both P conditions, was observed. Genotypic differences were evident, with DJ123 exhibiting increased organic resin-P concentrations in the rhizosphere compared to Nerica4. DJ123 demonstrated superior access to readily exchangeable organic P in the rhizosphere, highlighting a potential driver for its enhanced PAE. These findings emphasize the importance of genotype-specific strategies for optimizing P mobilization and acquisition in highly P-fixing soils.

## Introduction

High phosphorus (P) retention in agricultural soils is a major global challenge, particularly in regions where economic constraints limit the use of P fertilizers (Hedley et al. 1994). This challenge is especially pronounced in volcanic ash soils, such as Andosols, which are generally fertile but present low P availability, severely limiting crop production (Shoji et al. 1993b; Briceño et al. 2004). The high P sorption capacity of Andosols is caused primarily by the presence of iron (Fe) and aluminum (Al), which can be associated with non or poorly crystallized metal hydroxide complexes or crystalline (hydr-)oxides (Hiradate and Uchida 2004). These complexes have a large specific surface area and numerous reactive sites that can strongly adsorb phosphate ions (Hashimoto et al. 2012). Another characteristic of Andosols is their high organic matter content, which additionally contributes to increased P retention through the formation of Fe and Al organic matter complexes (Giesler et al. 2005; Hashimoto et al. 2012; Redel et al. 2016). Despite the low availability of P in these soils, the total P content is generally high, primarily due to the inherent P in the parent material and the substantial accumulation of legacy P from fertilizer application (Shoji et al. 1993a; Nobile et al. 2018). Given the financial constraints on P fertilizer application and the declining global reserves of rock phosphate, it is important from both an agronomic and an environmental perspective to acquire the soil’s existing P reserves, particularly in soils with a high P status or in soils where legacy P has accumulated (Doydora et al. 2020; Pavinato et al. 2020). Additionally, improving crop efficiency in the acquisition of freshly applied P fertilizer could provide a sustainable strategy to cope with limited P fertilizer availability.

One of the crops commonly cultivated on Andosols is upland rice (*Oryza sativa* L.), which is often grown in small-scale farming systems with low inorganic P fertilizer inputs (IRRI 2013). The selection of genotypes for increased P acquisition efficiency (PAE; P uptake per root surface area or root mass) could help solve the problem of low P fertilizer accessibility by increasing the soil P reserves accessible by the crop (Balemi and Negisho 2012; Shimamura et al. 2021). Yet, it is known that different upland rice genotypes exhibit varying efficiencies to acquire P, with some, such as DJ123, demonstrating higher PAEs than others, such as Nerica4 (Koide et al. 2013; Mori et al. 2016; Wissuwa et al. 2020; Mundschenk et al. 2024). However, the specific factors contributing to the elevated PAE in DJ123 are not yet fully understood. P mining strategies such as solubilization of recalcitrant P forms are likely to play a critical role in P-efficient genotypes. For example, previous research suggests that differences in P efficiency may result from differences in the acquisition of native soil P and that moderate P fertilizer application could enhance the uptake of native soil P resources (Mundschenk et al. 2024). Thus, we hypothesize that upland rice genotypes with contrasting PAEs can access different soil P forms or pools, which is most likely additionally influenced by the rate of P fertilizer application.

In terms of measuring soil P pools, sequential fractionation after Hedley et al. (1994) and later modified by Tiessen and Moir (2007) is most commonly used in agroecological research. In this method, P is trapped out of the soil via an anion exchange resin, and the P remaining in the soil is extracted with increasingly strong reagents and can be further categorized into inorganic P and organic P fractions (Cross and Schlesinger 1995; Johnson et al. 2003). The P fraction that binds to the exchange resin is readily exchangeable P, which is defined as labile P (Tiessen and Moir (2007) and is abbreviated as resin-P in the present study. Another labile P fraction can be obtained by extraction with NaHCO_3_ solution (Tiessen and Moir (2007) and is subsequently named NaHCO_3_-P. The P fraction extracted with the NaOH solution is defined as moderately labile P (Tiessen and Moir (2007) and is referred to below as NaOH-P. The P fraction that is defined as stable is extracted from the soil via a H_2_SO_4_ solution (Tiessen and Moir (2007) and is abbreviated as H_2_SO_4_-P below. With the exception of the resin P fraction, all of the other fractions listed here are typically measured for inorganic as well as organic P.

The labile P pool is considered to be easily turned over and contributes to the P available to plants and microbes (Cross and Schlesinger 1995; Johnson et al. 2003). In soil‒plant experiments, resin-P is typically quantified as inorganic resin-P, as it is regarded as the primary form of P available for plant uptake (Tiessen and Moir (2007). However, organic resin-P is rarely measured, despite evidence from previous research suggesting that it may contribute to short-term P uptake (Rubaek and Sibbesen 1993; McDowell et al. 2008). The moderately labile NaOH-P is considered to reflect P bound to Al-/Fe-(hydro)oxides, and the stable fraction predominantly reflects Ca-phosphates (Tiessen and Moir (2007). However, in soils rich in amorphous Fe and Al oxides and poor in calcium carbonate, such as Andosols, the stable P pool, which is commonly considered to be Ca-P extracted by strong acids, is more likely to reflect Fe- and Al-P (Gu et al. 2020).

To better understand the ability of rice genotypes with contrasting PAEs to access different P sources, we grew two upland rice genotypes in an Andosol with a low and sufficient P fertilizer supply. We investigated the inorganic and organic soil P pools at the beginning of the experiment (T_0_) and after harvest at 34 days after emergence (T_1_) via modified Hedley fractionation (Hedley et al. 1994; Tiessen and Moir (2007). To distinguish fertilizer from native soil P in the Hedley fractions, ^33^P-labeled fertilizer was applied at the beginning of the experiment. In this context, native soil P refers to the indigenous soil P and legacy P (non-radioactive P) present in the soil prior to the experiment, whereas"freshly applied"P refers to fertilizer P applied at the beginning of the experiment. We aimed to answer the following questions: (1) How does P fertilization and plant presence affect the distribution of fertilizer-P vs. native soil-P and organic- vs. inorganic-P within Hedley fractions? (2) Do genotypes varying in PAE access different amounts of fertilizer-P vs. native soil-P and organic- vs. inorganic-P in the rhizosphere?

## Materials and Methods

### Experimental Soil

A pot experiment was conducted with a P-deficient Andosol collected from an upland field site located in Tsukuba, Japan (36°03′09.6"N 140°04′39.2"E). The distribution of soil texture in the Andosol was as follows: 11% clay, 50% silt, and 39% sand. Further soil chemical properties of the used Andosol can be found in Mundschenk et al. (2024).

### Pot Experiment and Growth Conditions

We used a factorial design with two different upland rice (*Oryza sativa* L.) genotypes, two fertilizer treatments and two sampling dates. For each treatment combination, four replicates were grown in a completely randomized design. Genotype DJ123, which belongs to the *aus* subspecies of rice, is recognized for its high PAE, as observed in previous studies (Mori et al. 2016; Wissuwa et al. 2020; Mundschenk et al. 2024). As a contrasting genotype, Nerica4, which has a low PAE, was selected (Koide et al. 2013; Mori et al. 2016). Experiments were conducted in 1-L conical pots (inner upper diameter: 14 cm, height: 12 cm; Geli GmbH, Germany) filled with 700 g of soil sieved to pass through a 1 mm mesh. Prior to the start of the experiment, the soil was stored slightly moist in a dark room at approximately 15 °C. The soil was fertilized using nutrient solutions containing ammonium nitrate (NH_4_NO_3_), disodium phosphate (Na_2_HPO_4_)_,_ and potassium sulfate (K_2_SO_4_), selected based on the Yoshida solution formulation (Yoshida et al. 1972). Each nutrient solution was labeled with ^33^P using phosphoric acid (H_3_^33^PO_4_) (Hartmann Analytic GmbH, Brunswick, Germany). The amount of P introduced into the nutrient solution with the ^33^P was negligible due to high specific activity (S.A. in kBq ^33^P mg^−1^ P) of the purchased ^33^P (S.A.: low-P 2794.3 kBq ^33^P mg^−1^ P and high-P 137.7 kBq ^33^P mg^−1^ P). The fertilizer treatments differed in the amount of P applied: a high-P treatment (N:P:K, 100:70:100 mg kg^−1^) was compared with a low-P treatment (N:P:K, 100:3.5:100 mg kg^−1^). Fertilizer rates were determined based on a pre-experiment, in which the growth of genotypes under different P levels was evaluated. The low-P treatment included some P, as plant growth would have been inadequate without any P application. The nutrient solutions were mixed evenly into each batch of soil using a handheld mixer. Following the addition of the ^33^P-spiked nutrient solution to the soil, the soil was dried overnight at 65 °C, subsequently filled into the pots and watered to the maximum water holding capacity (max WHC: 70%, estimated according to DIN ISO 11274) the following day. One pregerminated seed per pot was subsequently placed into the soil. For the experiment, 6.6 MBq of ^33^P per pot were supplied, considering the short half-life of ^33^P (t_1/2_ 25.34 days). The day after fertilization and before planting, soil samples (T_0_) were taken. The second sampling was conducted at 34 DAE. The experiment was conducted in a climate chamber with a 14 h photoperiod at a light intensity of 300 µmol m^−2^s^−1^, a temperature of 30 °C during the day and 24 °C during the night and a relative humidity of 70%. The pots were maintained at a volumetric water content of 28–31% during plant growth (40–45% of the max WHC).

### Plant and Soil Analysis

At harvest, the shoots were cut, and the root system was carefully removed from the soil and gently shaken to remove loosely adhering soil. Loose roots in the remaining soil were subsequently picked with tweezers. Afterward, the assembled root samples with adhering soil were gently washed in deionized water, and the collected soil was defined as rhizosphere soil, while the remaining soil was considered bulk soil. The shoots and roots were oven-dried at 65 °C for 72 h for dry weight determination. All the dried plant samples were ground using a high-speed ball mill (Retsch M 400, Haan, Germany), and the subsamples were pressure digested in 2 ml of 64% HNO_3_ (König 2005**)**. The P content was determined using the modified molybdenum blue assay by Murphy & Riley (1962) as described by Tiessen and Moir (2007) in a microplate reader (SpectraMax High, Molecular Devices, USA). Total soil-P was assessed for soil samples taken at T_0_ and both bulk and rhizosphere soil. Samples of 0.05 g soil were pressure digested following the same procedure used for plant analysis. The P concentrations in the bulk and rhizosphere soil samples were calculated based on the exact masses of the recovered bulk soil and rhizosphere soil.

### P Sequential Fractionation

**Fig. 1:**
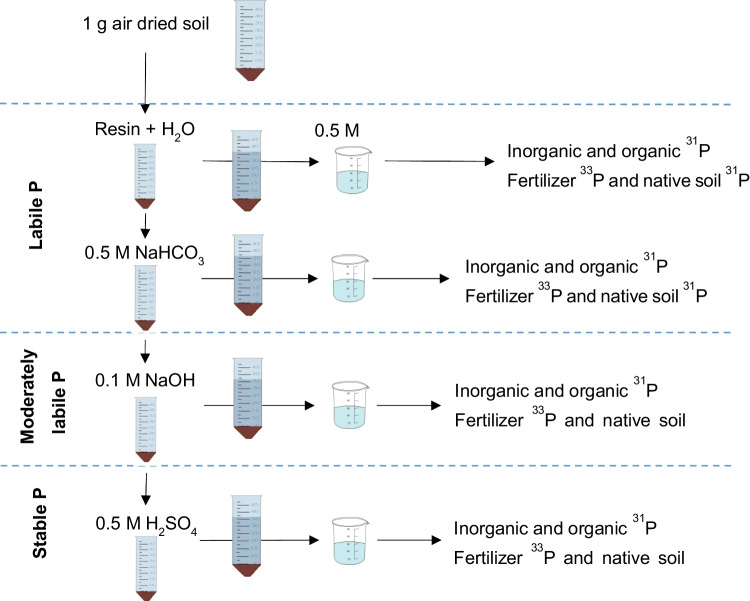
Hedley fractionation scheme adapted from Niederberger et al. (2015)

Sequential P fractionation was performed according to the method of Hedley et al. (1982) and modified by Tiessen and Moir (2007) on T_0_ soil samples and bulk and rhizosphere soil samples (34 DAE) (Fig. 1). To achieve a sufficient ^33^P signal, one gram of unground soil was placed into a 50 ml centrifuge tube and sequentially extracted with the following extractants: (1) 30 ml of deionized water with two resin strips (551642S, BDH-Prolabo, VWR International, Lutterworth, UK), followed by shaking the resin strips in 30 ml of 0.5 M HCl for 16 h (resin-P), (2) 30 ml of 0.5 M NaHCO_3_ at pH 8.5 (NaHCO_3_-P), (3) 30 ml of 0.1 M NaOH (NaOH-P) and (4) 0.5 M H_2_SO_4_ (H_2_SO_4_-P) (Fig. 1).

After every addition of extractant, the samples were continuously shaken for 16 h on a horizontal shaker, and the soil suspension was subsequently centrifuged at 4620 × *g* for 30 min. The supernatant was filtered through a 0.45 µm syringe filter and stored at 4 °C until ^33^P and P determination. Inorganic P from all Hedley extracts was determined via the molybdenum blue assay by Murphy and Riley (1962) as described above for plant and soil samples (Redel et al. 2016, 2019). The total P in resin-P, NaHCO_3_-P, NaOH-P and H_2_SO_4_-P was measured via ICP‒OES (ICP-iCAP 6300 DUO, Thermo Fisher Scientific GmbH, USA), and the organic P was then calculated from the difference between the total P and inorganic P of the respective fraction (Vu et al. 2010; Do Nascimento et al. 2015). The sum of P, referred to as total P, was calculated as the sum of each individual fraction.

### ^33^P Determination

The ^33^P activity in all pressure-digested plant and soil extracts was assessed through liquid scintillation counting. One ml of the extract was mixed with 10 ml of scintillation cocktail (Rotiszint eco high, Roth, Karlsruhe, Germany) and subsequently measured via a liquid scintillation counter (Tri-Carb® 2800 TR, Perkin Elmer, Germany). For the Hedley extracts, 2 ml of each extract was mixed with 15 ml of the following scintillation cocktails to ensure precise measurements (with low luminescence): (1) resin-P and H_2_SO_4_-P were measured in Hionic Fluor (Perkin Elmer, Waltham, USA), and (2) NaHCO_3_-P and NaOH-P were measured in Rotiszint eco plus. This step was necessary because the varying strengths of the acids and bases interact differently with the scintillation cocktail. To account for the interval between the setup of the experiments and measurement, the ^33^P signal was corrected for the ^33^P t_1/2_ of 25.34 days on the reference day of each radioactive source [Eq. 1], with λ representing the decay constant.

1 $${t}_\frac{1}{2}=\frac{\text{ln}\left(2\right)}{\lambda }$$

The specific ^33^P activity of a sample (soil or Hedley extracts) was calculated according to IAEA (2001):

2 $$\text{S.A.} \left[{\text{kBq}} {}^{33}{\text{P}} {\text{mg}}^{-1}\text{ P}\right]\text{=} \frac{{}^{33}{\text{P}}\left[{}^{33}{\text{P}} {\text{mg}}^{-1}\text{ soil}\right]}{{\text{P}} \left[\text{mg P} {\text{mg}}^{-1}\text{ soil}\right]}$$

The amount of P derived from fertilizer was calculated according to Eq. (4) (Dorahy et al. 2007):

3 $$\mathrm P\;\mathrm{derived}\;\mathrm{from}\;\mathrm{fertilizer}\;\left[\mathrm{mg}\;\mathrm P\;\mathrm{fraction}^{-1}\right]=\frac{{}^{33}\mathrm P\;\mathrm{content}\;\mathrm{fraction}\;\left[\mathrm{kBq}{}^{33}\mathrm P\;\mathrm{fraction}^{-1}\right]}{\mathrm S.\mathrm A\;\mathrm{of}\;\mathrm{labeled}\;\mathrm{fertilizer}\;\left[\mathrm{kBq}{}^{33}\mathrm P\;\mathrm{mg}^{-1}\mathrm P\right]}$$

The quantity of P originating from native soil-P was calculated by subtracting the amount of P derived from fertilizer from the total P content of each fraction. Note that the method used allows for the distinction of native versus fertilizer-derived P but not further differentiation into organic/inorganic native versus fertilizer-derived P.

The amount of fertilizer-P uptake was calculated according to Eq. 5 (Dorahy et al. 2007):

4 $$\mathrm P\;\mathrm{uptake}\;\mathrm{from}\;\mathrm{fertilizer}\;\mathrm P\left[\mathrm{mg}\;\mathrm P\;\mathrm{plant}^{-1}\right]=\frac{{}^{33}\mathrm P\;\mathrm{up}\;\mathrm{take}\;\mathrm{plant}^{-1}}{\mathrm S.\mathrm A\;\mathrm{of}\;\mathrm{labeled}\;\mathrm{fertilizer}\;\left[\mathrm{kBq}{}^{33}\mathrm P\;\mathrm{mg}^{-1}\mathrm P\right]}$$

The quantity of native soil P uptake was calculated by subtracting the amount of P derived from fertilizer from the plant P uptake.

### Statistical Analysis

All analyses were conducted using the statistical software R (version 4.1.3; R Core Team, USA). The effects of genotype (DJ123 and Nerica4), P fertilizer (low-P or high-P) and the interaction of the two treatments were analyzed using a two-way analysis of variance (ANOVA) on all the measured data from the T_0_, bulk and rhizosphere soils. Outliers were identified using descriptive statistics and boxplots, and one replicate from the low-P treatment was removed as a result. The normal distribution and variance homogeneity of the residuals were tested prior to analysis using the Shapiro‒Wilk test, the Levene test, and the R `car´ package, respectively. The significance of the differences between genotypes and P treatments was assessed using Tukey´s honestly significant difference (HSD) test with a threshold of p ≤ 0.05. Paired t-tests were used to compare the relative and absolute changes in P concentrations across different soil-P fractions between initial soil and bulk soil as well as rhizosphere soil, with p ≤ 0.05 considered significant. The relative and absolute changes were also analyzed using a one-way ANOVA to assess significant differences between the two P treatments and between genotypes within each treatment. The means are presented ± the standard error of the means (± SE).

## Results 

### Initial Distribution of Soil P Fractions Following Fertilization

Following the application of fertilizer-P at T_0_, the total P concentration in the soil ranged from 912.4 to 964.0 mg kg^−1^ under low- and high-P conditions, respectively. Note that the total P concentration is the sum of the inorganic and organic P concentrations and includes native soil P and recently applied fertilizer-P. Therefore, the total P concentration is identical for total inorganic and organic P and for total fertilizer and native soil-P.

**Fig. 2:**
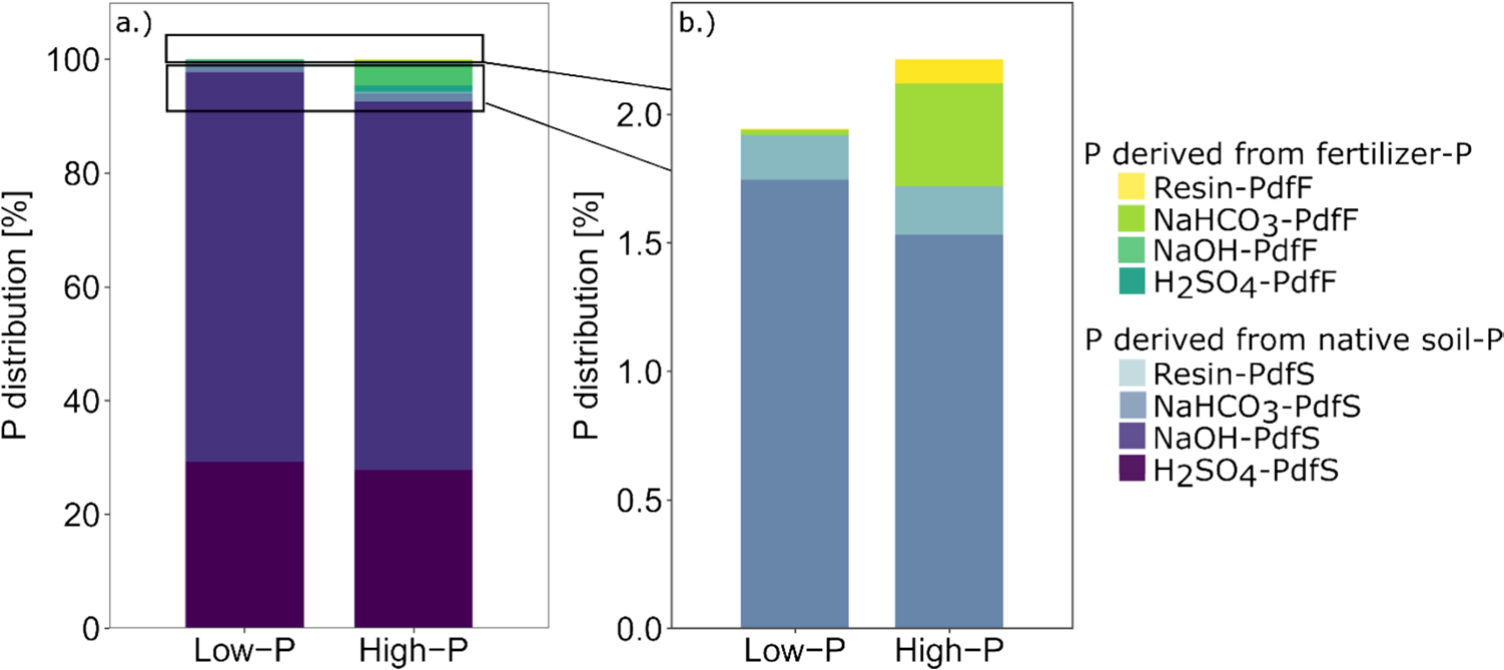
Hedley fractions of different soil P pools expressed as a percentage of total soil P originating from fertilizer-derived P (PdfF) and native soil-derived P (PdfS) in the low- and high-P treatments directly after fertilization (a.). Detailed view of the distributions of the labile P fractions and P pools (PdfF and PdfS) in the low- and high-P treatments at T_0_ (b.)

At T_0_, ^33^P was detected in all Hedley fractions, and the measured ^33^P activities indicated that most applied fertilizer-P entered the NaOH-P fraction (72.4% and 72.0%), followed by H_2_SO_4_-P (19.8% and 19.4%), NaHCO_3_-P (6.5% and 7.0%) and resin-P fractions (1.3% and 1.6%) under low- and high-P conditions, respectively (Fig. 2).

**Fig. 3:**
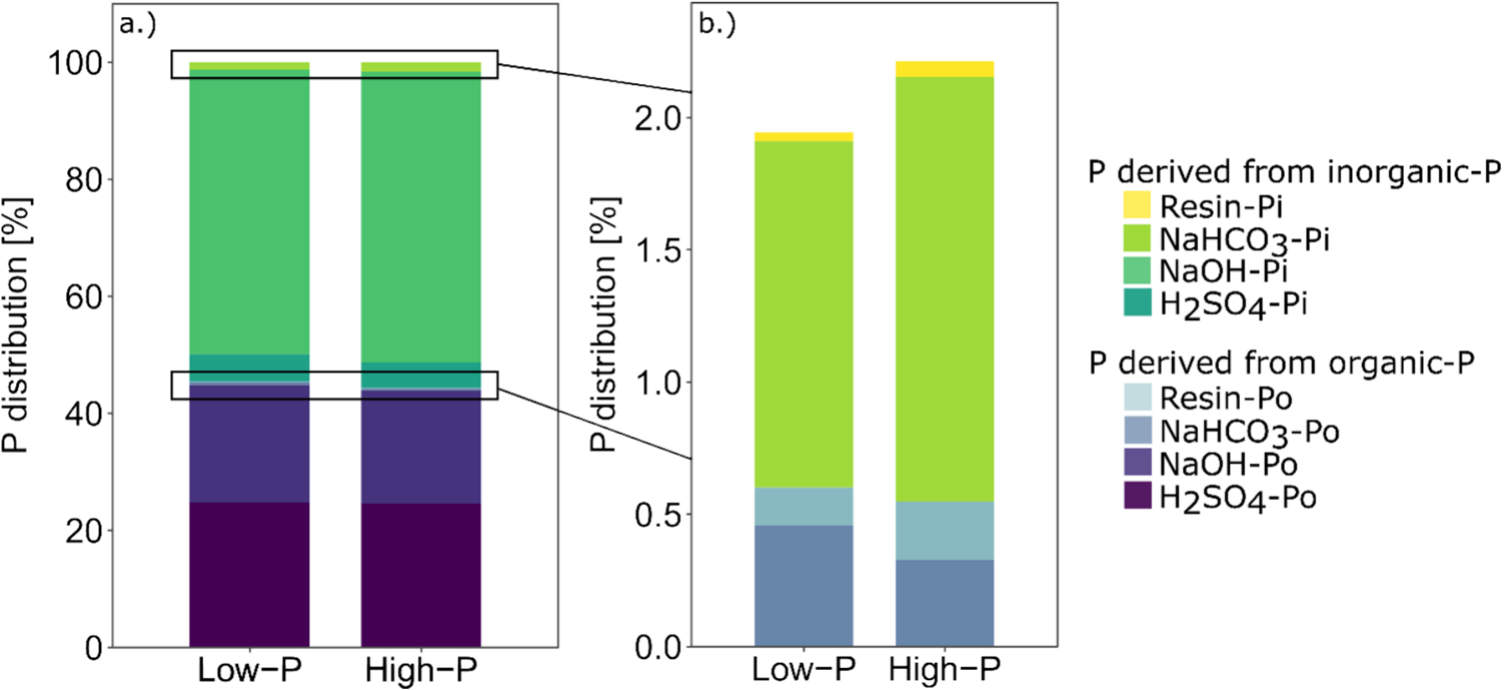
Hedley fractions of different soil P pools expressed as a percentage of total soil P originating from inorganic (Pi) and organic (Po) P in the low- and high-P treatments directly after fertilization (a.). Detailed view of the distribution of labile P fractions and P pools (Pi and Po) in the low- and high-P treatments at T_0_ (b.)

**Fig. 4:**
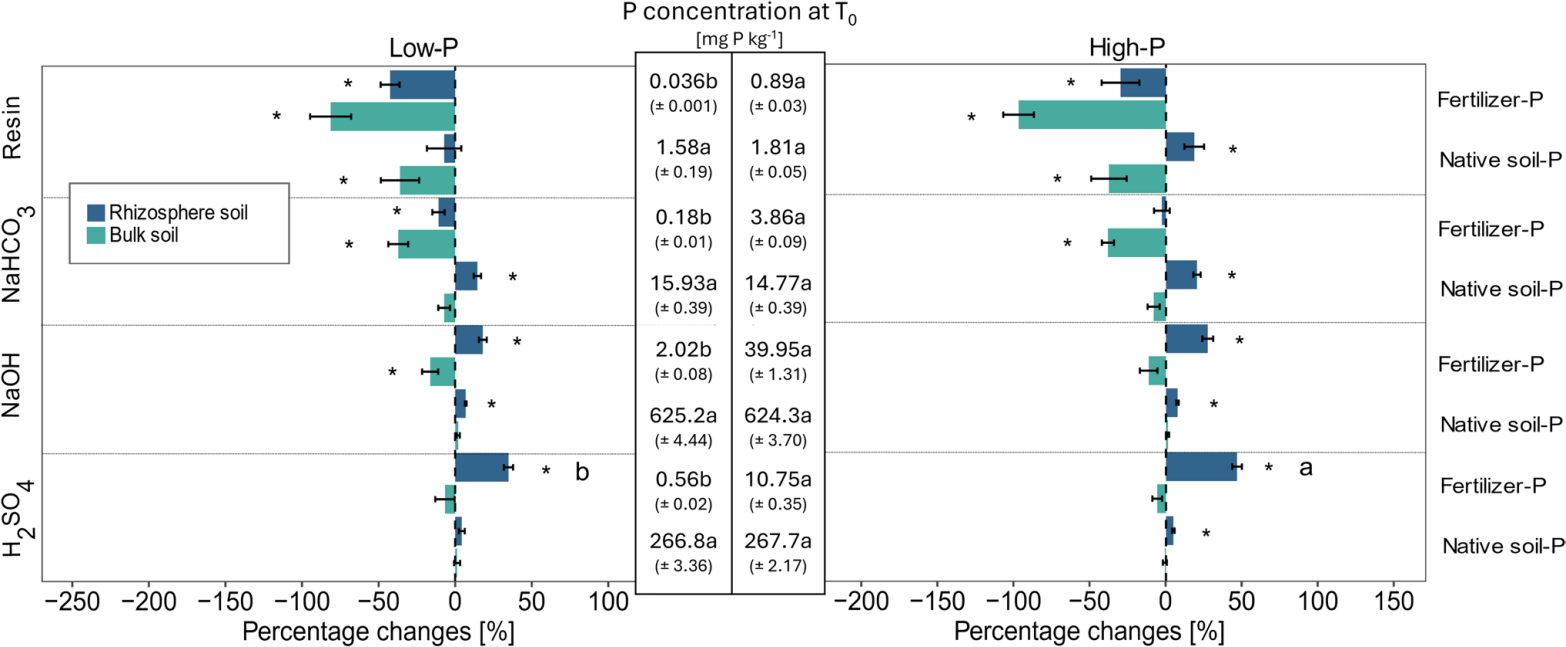
Percent difference in bulk and rhizosphere soils compared with their initial values at T_0_ (T_0_ P concentrations according to the low- and high-P treatments are shown in the middle) for P concentrations of fertilizer- and native soil-P in the four Hedley fractions under low- and high-P conditions, respectively. Variation is given as ± SE. * Indicates significant differences from T_0_ per P fraction (p < 0.05, paired t-test). Different letters indicate significant differences between the low- and high-P treatments in terms of the percentage difference for each P fraction and inorganic and organic P pools (p < 0.05, One-way ANOVA, n = 7–8)

The most labile P form, resin-P, comprised approximately 0.18% to 0.28% of the total P, whereas the other labile P fraction, NaHCO_3_-P, made up 1.77% to 1.93% of the total P under low- and high-P conditions (Fig. 2b and 3b). The largest share of P, approximately 68.8% to 68.9%, was found in the moderately available NaOH-P fractions (Fig. 2a and 3a). H_2_SO_4_-P, regarded as the most stable P fraction, accounted for approximately 29.3% to 28.9% of the total P under both low-P and high-P conditions at T_0_. In all the fractions, a significantly greater concentration of fertilizer-P was observed in the high-P treatment than in the low-P treatment (Fig. 4—absolute concentrations at T_0_). The concentrations of native soil P in the different P fractions were not affected by the two fertilizer treatments (Fig. 4).

**Fig. 5:**
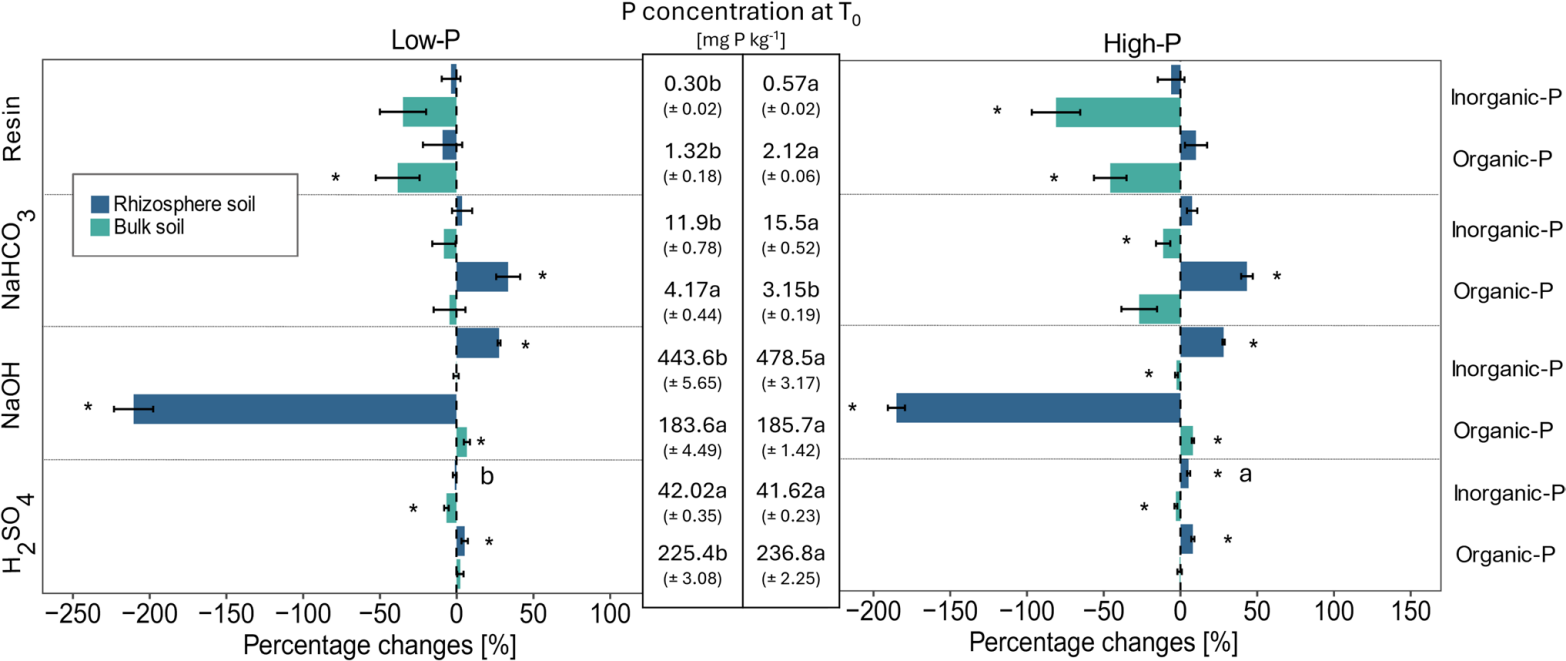
Percent difference in bulk and rhizosphere soils compared with their initial values at T_0_ (T_0_ P concentrations according to the low- and high-P treatments are shown in the middle) for P concentrations of inorganic and organic P in the four Hedley fractions under low- and high-P conditions, respectively. Variation is given as ± SE. * Indicates significant differences from T_0_ per P fraction (p < 0.05, paired t-test). Different letters indicate significant differences between the low- and high-P treatments in terms of the percentage difference for each P fraction and inorganic and organic P pools (p < 0.05, One-way ANOVA, n = 7–8)

On average, 55% (± 0.6%) of the total P was identified as inorganic-P, whereas 45% (± 0.6%) was present as organic-P (Fig. 3a.). These proportions were comparable for both P treatments, and inorganic and organic P were distributed similarly among the different P pools under both low- and high-P conditions at T_0_ (Fig. 3a.). On average, 89% of the total inorganic P pool, constituting 55% of the overall P, was found in the NaOH-P fraction under both low- and high-P conditions (Fig. 5 – absolute P concentrations at T_0_). This fraction was followed by the H_2_SO_4_-P fraction (ranging from 7.8% to 8.4%), the NaHCO_3_-P fraction (2.4% to 2.9%) and the resin-P fraction (0.06% to 0.11%) (Fig. 5) under low- and high-P conditions, respectively. In terms of organic P, 54.4 to 55.4% of the total organic P was associated with the H_2_SO_4_ fraction, followed by the NaOH fraction (44.3% to 43.4%), NaHCO_3_-P fraction (ranging from 0.7% to 1.0%), and resin-P fraction (0.3% to 0.5%) (Fig. 5).

At T_0_, the higher application of fertilizer-P in the high-P treatment led to a significant increase in P concentrations in nearly all Hedley fractions (Fig. 5). The most notable relative increase was observed in the resin-P fraction, which exhibited a 67% increase under high-P conditions compared to the low-P treatment (p < 0.05). Within this fraction, there was a 93% increase (p < 0.05) in the inorganic-P pool and a 61% increase (p < 0.05) in the organic-P pool. This was followed by the NaHCO_3_-P (+ 16%, p < 0.05) fraction, where a 30% increase was noted in the inorganic NaHCO_3_-P pool (p < 0.05), while organic P decreased by 24% (p < 0.05). The inorganic NaOH-P fraction increased by 6% (p < 0.05), and the organic H_2_SO_4_-P fraction increased by 4% (p < 0.05). No significant difference was observed between the two P treatments in the organic NaOH or inorganic H₂SO₄ fractions. However, when the total increase in concentration of each fraction after fertilization at T_0_ was considered, the largest increase in P concentration was observed in the NaOH-P fraction (+ 37.0 mg kg^−1^, p < 0.05), followed by the H_2_SO_4_-P fraction (+ 11.1 mg kg^−1^, p < 0.05), NaHCO_3_-P fraction (+ 2.5 mg kg^−1^, p < 0.05) and resin-P fraction (+ 1.1 mg kg^−1^, p < 0.05) (Fig. 4).

### Plant Effects on P Distribution in Bulk and Rhizosphere Soils

Compared to that in the low-P treatment, plant P uptake in the high-P treatment increased significantly, which was driven primarily by increased fertilizer P uptake (Table 1). Additionally, increased P fertilization led to greater uptake of native soil P. On the basis of the moderate plant tissue P concentrations analyzed at the end of the experiment (~ 1.67 mg P g^−1^), the term high-P treatment in this study refers to a moderate P treatment (Yoshida 1981; Hedley et al. 1994).

**Table 1:** Quantity of fertilizer (QPdfF) and native soil-derived P (QPdfS) uptake per plant 34 days after emergence per P fertilizer treatment, adapted from Mundschenk et al. (2024)

		Low-P		High-P	
QPdfF	[mg fertilizer P uptake plant^−1^]	0.011	b	1.218	a
		± 0.002		± 0.144	
QPdfS	[mg native soil-P uptake plant^−1^]	0.166	b	0.544	a
		± 0.024		± 0.085	

Values are the means ± SEs. Different letters indicate significant differences between the genotypes in the low- or high-P treatment (p < 0.05; Tukey’s HSD, One-way ANOVA, n = 7‒8).

Plant growth significantly altered the distribution of P among various P fractions in the bulk and rhizosphere soils. The total P in the bulk soil ranged from 927.0 to 963.2 mg kg-^1^, while in the rhizosphere soil, it ranged from 974.1 to 1058.0 mg kg-^1^ under low- and high-P conditions, respectively (Suppl. Tables S1 and S2). No significant changes in total P were observed in the bulk soil under either low- or high-P conditions upon plant growth (Suppl. Tables S1 and S2). However, in the rhizosphere, the total P concentration increased significantly under both treatments, with a relative increase of 6.3% under low-P and 8.8% under high-P conditions compared to the initial soil (Suppl. Tables S1 and S2).

#### Fertilizer and Native Soil P Dynamics in Bulk and Rhizosphere Soil

After 34 days, the relative differences in P derived from fertilizer and native soil were consistent between the bulk and rhizosphere soils. Similar trends were observed under both low- and high-P conditions compared to the initial soil (Fig. 4). Significant differences in the relative changes between the two P treatments were observed only in the rhizosphere, specifically in the fertilizer-P increase within the H₂SO₄-P fraction (Fig. 4).

When the absolute changes in the Hedley fractions with respect to plant growth were considered, significant differences between the low- and high-P treatments were observed in the bulk and rhizosphere soils (Suppl. Table S2). In the bulk soil, total fertilizer-P decreased upon plant growth, whereas in the rhizosphere, plant growth resulted in increased fertilizer-P concentrations under both P conditions (Suppl. Table S2). Under high-P conditions, a greater decrease in fertilizer-P was observed in the resin and NaHCO_3_-P fractions in the bulk soil than in the low-P treatment (Suppl. Table S2). Moreover, fertilizer-P in the NaOH- and H_2_SO_4_-P fractions increased significantly in the rhizosphere under high-P conditions compared to low-P conditions (Suppl. Table S2). A significant increase in native soil resin-P was observed in the rhizosphere only under high-P conditions, whereas no change was detected under low-P conditions.

In the bulk soil, the largest relative decrease in fertilizer-P relative to that in the initial soil was observed in the resin P fraction, with decreases of −81.3% and −96.3% (p < 0.05) under low- and high-P conditions, respectively (Fig. 4). This was followed by decreases in fertilizer-P in the NaHCO₃-P fraction (−37.2% and −37.9% (p < 0.05)), the NaOH-P fraction (−16.3% and −11.3%), and the H₂SO₄-P fraction (−15.5% and −12.1%) under low- and high-P conditions, respectively (Fig. 4).

While fertilizer-P generally decreased in the bulk soil, the relative concentrations of native soil-P remained largely unaffected by plant presence, except in the resin-P fraction, where native soil-P decreased by −36% under low-P conditions and by −37.3% under high-P conditions (Fig. 4).

In the rhizosphere, fertilizer-P decreased significantly, by −42.5% and −10.9%, in the resin-P and NaHCO_3_-P fractions, respectively, under low-P conditions. Under high-P conditions, a significant decrease of −29.7% in fertilizer-P was observed in the resin-P fraction, whereas fertilizer-P remained unchanged in the NaHCO_3_-P fraction (Fig. 4). In contrast, fertilizer-P increased in the moderately labile and stable P fractions in the rhizosphere soil under low- and high-P conditions. Native soil P increased relatively in all Hedley P fractions in the rhizosphere under high-P conditions, whereas under low-P conditions, increases were observed only in the NaHCO_3_-P and NaOH-P fractions (Fig. 4).

Once more, when absolute changes in P concentrations were considered, the influence of plant presence was greater in the rhizosphere than in the bulk soil (Suppl. Table S2). The most notable changes were observed in the NaOH-P fraction of both fertilizer- and native soil-P. The concentration of fertilizer-P increased by 15.5 mg kg⁻^1^ under high-P conditions, whereas for native soil-P, it increased by 46.1 mg kg⁻^1^ under low-P conditions and by 51.19 mg kg⁻^1^ under high-P conditions.

#### Inorganic and Organic P Dynamics in Bulk and Rhizosphere Soil

The relative changes in inorganic and organic P resulting from plant growth showed similar trends under both P levels (Fig. 5). A significant difference between the two P treatments was observed in the absolute changes in the inorganic resin-P concentration in the bulk soil, with a greater decrease of –0.244 mg P kg⁻^1^ under high-P conditions compared to –0.068 mg P kg⁻^1^ under low-P conditions (Suppl. Table S4). No significant differences between the low- and high-P treatments were detected in the remaining P pools across the various P fractions in either the bulk or rhizosphere soil.

In the bulk soil, the labile P pool concentrations were affected mainly under high-P conditions, where plant growth significantly decreased the inorganic and organic resin-P and the inorganic NaHCO₃-P by −81.1%, −45.8%, and −11.3%, respectively (Fig. 5). Under low-P conditions, only the organic resin-P decreased significantly (−38.3%). The total organic P pool in the bulk soil increased by 4.3% under low- and 3.2% under high-P conditions, mainly due to organic NaOH-P. Under high-P conditions, a significant decrease of −2.6% in the inorganic NaOH-P fraction was observed following plant growth. A small but significant decrease in the inorganic H₂SO₄-P pool was also observed, with reductions of −6.5% and −3.0% under low- and high-P conditions, respectively.

Following plant growth, no significant changes were observed in the rhizosphere in the labile inorganic and organic P fractions, apart from a significant increase in organic NaHCO_3_-P under low- and high-P conditions (Fig. 5). For most of the other P pools, we observed increases in the rhizosphere under both low- and high-P conditions, except for the inorganic H₂SO₄-P pool under low-P conditions. The largest increase in P was observed in the inorganic NaOH-P fraction (27.8% under low-P and 28.1% under high-P conditions). This increase was accompanied by a significant decrease in the organic NaOH-P fraction, which decreased by −210.5% under low-P conditions and −185.1% under high-P conditions.

Regarding absolute changes in the P fractions in response to plant presence, the greatest changes were observed in the rhizosphere, particularly in the inorganic and organic NaOH fractions (Suppl. Table S4). The inorganic P increased by 170.8 and 187.1 mg kg⁻^1^, while the organic P pool decreased by −120.4 and −124.2 mg kg⁻^1^ under both P conditions.

### Genotypic Variations in P Changes between Bulk and Rhizosphere Soil

Under low-P conditions, fertilizer-P uptake was similar between the genotypes, but under high-P conditions, DJ123 took up significantly more fertilizer-P than Nerica4 (Table 2). Notably, native soil P uptake was significantly greater for DJ123 than for Nerica4 under both P treatments (Table 2).

**Table 2:** Quantity of fertilizer (QPdfF) and native soil-derived P (QPdfS) uptake per plant at 34 days after emergence, adapted from Mundschenk et al. (2024)

		Low-P				High-P			
		DJ123		Nerica4		DJ123		Nerica4	
QPdfF	[mg fertilizer P uptake plant^−1^]	0.011	a	0.010	a	1.568	a	0.868	b
		± 0.002		± 0.003		± 0.098		± 0.075	
QPdfS	[mg native soil-P uptake plant^−1^]	0.210	a	0.107	b	0.763	a	0.325	b
		± 0.019		± 0.012		± 0.030		± 0.019	

Values are the means ± SEs. Different letters indicate significant differences between the genotypes in the low- or high-P treatment (p < 0.05; Tukey’s HSD, One-way ANOVA, n = 3–4).

**Fig. 6:**
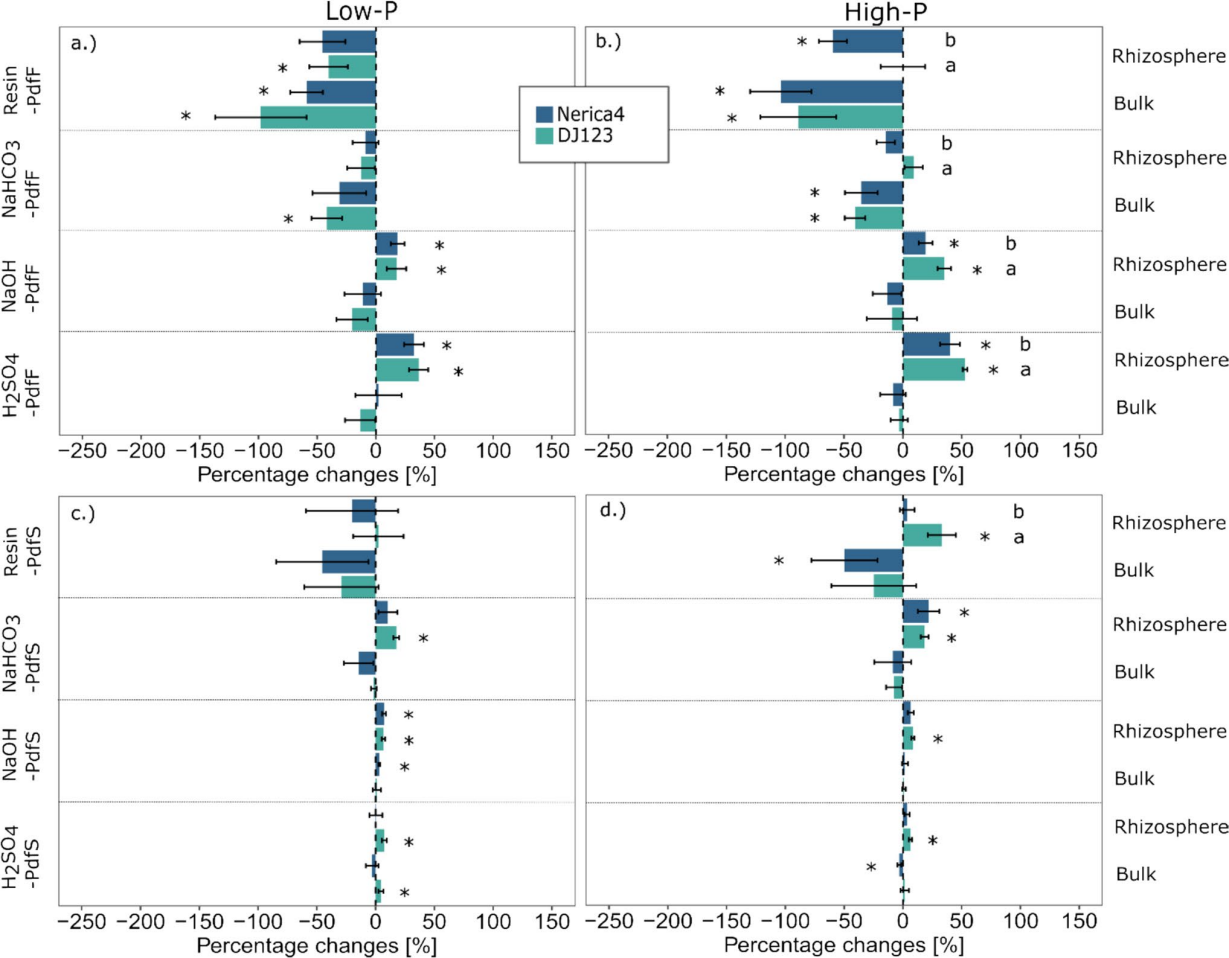
Percent difference between bulk and rhizosphere soils and initial soils (T_0_) in terms of individual P concentrations of fertilizer-derived P (a. and b.; PdfF) and native soil-derived P (c. and d.; PdfS) in the four Hedley fractions under low- and high-P conditions, respectively. Variation is given as ± SE. * Indicates significant differences from T_0_ per P fraction (p < 0.05, paired t-test). Distinct letters indicate significant differences between genotypes (p < 0.05, Tukey’s HSD, One-way ANOVA, n = 3–4) in terms of percentage difference from T_0_ in the different fertilizer treatments

The relative differences in fertilizer and native soil P followed a similar trend between the low- and high-P treatments, with no differences between genotypes under low-P conditions (Fig. 6). Significant differences in the relative change in fertilizer-P concentration were found mainly in the rhizosphere under high-P conditions (Fig. 6b). The fertilizer-P in the resin-P fraction in the rhizosphere of Nerica4 was reduced by −59.3%, whereas no change was observed for DJ123 (Fig. 6b). The opposite effect was found for native soil P in the resin-P fraction, which increased by 33.3% in DJ123, while no significant change was observed in Nerica4 (Fig. 6d). A significant genotypic difference was evident in the relative change in fertilizer-derived NaHCO_3_-P in the rhizosphere (Fig. 6b). However, the relative change in fertilizer NaHCO_3_-P in the rhizosphere soil was not statistically significantly different from that in the initial soil for either genotype. For the moderately labile fertilizer-P in the NaOH-P fraction, DJ123 showed a significantly greater increase (35.2%) than did Nerica4 (19.3%). This trend was even more pronounced for fertilizer-P in the stable H_2_SO_4_-P fraction, where DJ123 showed a 52.8% increase compared to 40% in Nerica4 (Fig. 6b). The fertilizer and native soil P concentrations per Hedley fraction for each genotype and P treatment are provided in Supplementary Table S5.

**Fig. 7:**
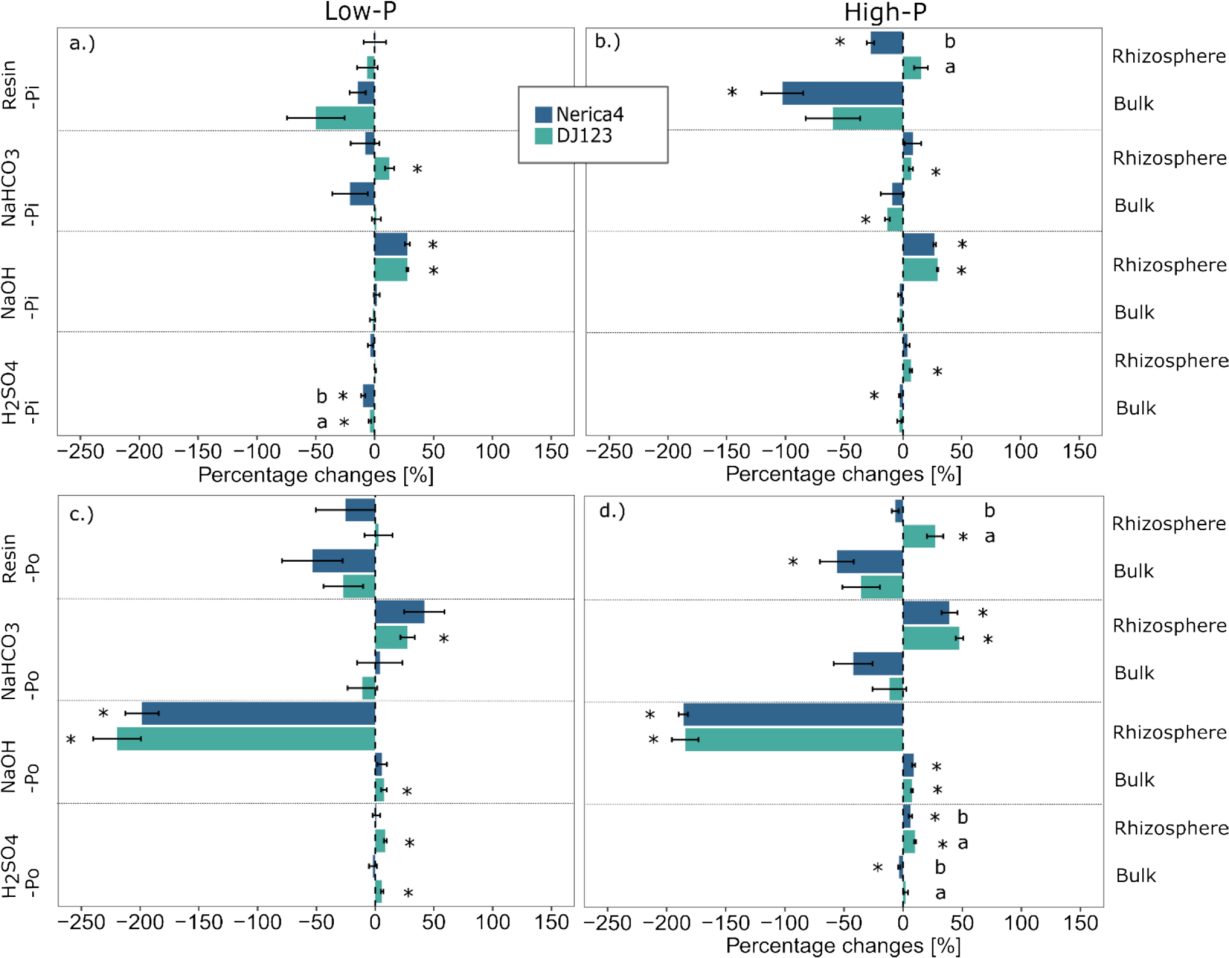
Percent difference in bulk and rhizosphere soils compared with their initial values at T_0_ for P concentrations of inorganic (a. and b.; Pi) and organic P (c. and d.; Po) in the four Hedley fractions under low- and high-P conditions, respectively. Variation is given as ± SE). * Indicates significant differences from T_0_ per P fraction (p < 0.05, paired t-test). Distinct letters indicate significant differences between genotypes (p < 0.05, Tukey’s HSD, One-way ANOVA, n = 3–4) in terms of percentage difference from T_0_ in the different fertilizer treatments

Genotypic differences in inorganic and organic P changes were evident mainly in the high-P treatment (Fig. 7). These genotypic differences were observed in both the inorganic and organic resin-P pools in the rhizosphere (Fig. 7b and d). Inorganic resin-P was not significantly affected by the growth of DJ123, whereas it significantly decreased by 27.6% in the rhizosphere of Nerica4 (Fig. 7b). A different trend was observed for the organic resin-P fraction, which increased by 26.9% in the rhizosphere of DJ123 (Fig. 7d). The inorganic and organic soil P concentrations per Hedley fraction for each genotype and P treatment are provided in Supplementary Table S6.

## Discussion

High P fertilization increased organic P and inorganic P one day after fertilization, with fertilizer-P entering all four Hedley fractions. This indicates that the easily available P from the inorganic fertilizer was rapidly adsorbed onto the soil matrix. Furthermore, the results suggest a rapid microbiological transformation into organic P forms. While only low amounts of fertilizer-P were found in the labile P fractions (8.2%), large amounts were recovered in the NaOH-P fraction (72.2%) and in the H_2_SO_4_ fraction (19.6%) (Fig. 4). This is in agreement with previous studies, where NaOH-P was identified as the primary sink for fertilizer-P (Li et al. 2008a, b; Ciampitti et al. 2011; Touhami et al. 2020).

The total P concentrations of the studied Andosol were comparable to those of the top 0–20 cm of an Andosol from Chile under grassland (1019.6 mg P kg⁻^1^) (Velásquez et al. 2016), to the mean value of Andosols (903 mg P kg^−1^) analyzed in a meta-analysis by Yang and Post (2011) and to highly weathered soils from rice fields in Madagascar (960 mg kg⁻^1^) (Nishigaki et al. 2019). In Andosols, the highest P concentrations are present in the moderately labile and stable P fractions, whereas the labile P concentration is extremely low (Takeda et al. 2009; Nishigaki et al. 2019). Similar to Nishigaki et al. (2019), the largest share of total P was found in the inorganic NaOH-P fraction in our study (Fig. 3). Andosols are characterized by a high content of organic P that can constitute between 30–82% of the total P in the top layer of soils (Redel et al. 2016). With 45% organic P, the tested Andosol fell within this range. The transformation from inorganic to organic P in Andosols is considered to be a significant sink for applied P fertilizer and therefore plays a crucial role in P retention (Escudey et al. 2001). This is confirmed by our study, where more than 54% of the total organic P was found to be in the stable H_2_SO_4_-P fraction, followed by the NaOH-P fraction, which contained more than 43% of the total organic P.

Resin-P is considered readily exchangeable or plant-available inorganic P (Sibbesen 1978) that enters the solution by dissolution or desorption from the soil solid phase or through the mineralization of organic P (Hartono et al. 2006; Tiessen and Moir 2007). As in our study, the measurement of inorganic P is therefore most commonly conducted via the method of Murphy and Riley (1962), which considers the measured P as orthophosphate or molybdate reactive inorganic P (Sibbesen 1978; Buehler et al. 2002; Cheesman et al. 2010; Vu et al. 2010; Nishigaki et al. 2019). In contrast, few studies have measured total resin-P concentrations to calculate organic P levels (McDowell et al. 2008; Cheesman et al. 2010). Total P is typically determined either by using ICP analysis or by digesting the Hedley extract (e.g., acid digestion) followed by measurement with e.g. the molybdate blue method (McDowell et al. 2008; Vu et al. 2010). For the resin P fraction of the low-P fertilization treatment in our study, the total amount of fertilizer P calculated on the basis of ^33^P activity could be completely detected by the molybdenum blue method. In contrast, for the resin P fraction of the high-P treatment, more P from fertilizer (0.89 mg P kg^−1^, fertilizer-P, Fig. 4) was detected on the basis of the ^33^P activity than could be measured via the molybdenum blue method (0.57 mg P kg^−1^, inorganic P, Fig. 5) at T_0_. To address this discrepancy, we reanalyzed the resin extracts via ICP and identified a substantial portion of P within the organic or condensed inorganic P pool. These findings suggest that within the short period between ^33^P application and sampling, some of the P from fertilizer had already been transformed into organic P or condensed inorganic P compounds, which were not detectable via the molybdenum blue method. This assumption is supported by the fact that in the resin P fraction of the high-P treatments, almost four times as much organic P as inorganic P was found at T_0_. The effect was most pronounced at T_0_ and to a lesser extent in the bulk and rhizosphere soils, indicating that parts of the resin-P were present as organic resin-P. Previous studies also demonstrated that some organic and condensed inorganic P compounds were fully recovered by anion resin membranes, whereas other P compounds were only partly recovered (Cheesman et al. 2010). Our results indicate that (1) large amounts of the P recovered from the resin strips were organic and/or condensed inorganic P, (2) under high-P fertilization, a substantial amount of fertilizer-P was found in a presumably readily available organic or complexed inorganic P form one day after fertilizer addition, and (3) the amount of fertilizer-P in the organic resin-P fraction decreased during 34 days of plant growth.

As demonstrated in the study conducted by Mundschenk et al. (2024), the application of fertilizer P increased the uptake of native soil P in the high-P treatment. Furthermore, native soil-derived P constituted a significant P source under low-P conditions, whereas under high-P conditions, the plants exhibited greater P fertilizer uptake.

We observed a decrease in most labile P forms for both fertilizer-P and native soil-P in the bulk soil, except for the native soil-P in the NaHCO_3_-P fraction, which remained stable under both P conditions (Fig. 4, Suppl. Table S2). Additionally, under high-P conditions, the inorganic and organic labile P fractions decreased, apart from the organic NaHCO_3_-P fraction, which remained unaffected by plant growth (Fig. 4, Suppl. Table S2). These results indicate that the labile P fractions were mobilized in the bulk soil and either moved to the rhizosphere or were converted by soil processes into other P fractions. The bulk soil is commonly defined as the soil not directly influenced by plant roots, in contrast to the rhizosphere, which Hiltner (1904) described as ‘the soil influenced by roots’. However, this distinction, particularly when sampling methods that define the rhizosphere as only the soil adhering to roots, has certain limitations. As reviewed by York et al. (2016), the rhizosphere may extend beyond the narrow zone sampled via this method, suggesting that the considerable bulk soil could still be influenced by plant roots, particularly in pot experiments with narrow pots. In such cases, the bulk soil may simply represent soil located further from the root system rather than truly root-unaffected soil. This methodological limitation may underestimate the rhizosphere effect.

The P concentration in the rhizosphere is commonly considered to be depleted compared to that in the bulk soil, as plant P uptake is assumed to be faster than P replenishment from the bulk soil through diffusion (Cross and Schlesinger 1995; Hinsinger 2001; Hinsinger et al. 2011). Depletion is most likely to occur in the labile P fractions resin-P and NaHCO_3_-P, which are recognized as the most plant-available forms of P (Cross and Schlesinger 1995; Johnson et al. 2003; Tiessen and Moir 2007). However, we found that native soil P and organic P increased in the NaHCO_3_-P fraction under both P treatments. Li et al. (2008a, b) reported a 61% increase in organic NaHCO_3_-P in the rhizosphere of monocropped bean grown in a chromic Cambisol and attributed this to a transformation of inorganic P rather than hydrolysis of organic P due to increased microbial activity. In contrast, in our study, a significant decline in fertilizer-P was observed within the resin-P fraction across both P treatments, whereas changes in the inorganic P pool within the labile fractions were not detected. Other studies reported a depletion of labile P in the rhizosphere of rice grown on an Ultisol (Li et al. 2008b) or of maize and lupin grown on an Andosol (Dissanayaka et al. 2015). In addition, in a meta-analysis, Liu et al. (2022) reported a general decrease in available P of 12% in rhizosphere soil compared to that in bulk soil. However, a number of studies have reported increased P concentrations in the rhizosphere soil compared to those in the bulk soil (Chen et al. 2002; Betencourt et al. 2012; Sun et al. 2020; Ogola et al. 2023). The uptake-driven P depletion in the rhizosphere could be counteracted by soil P mobilization caused by mineralization processes occurring simultaneously with P depletion, diminishing the dominant effect of P depletion through the plant (Devau et al. 2010, 2011). Furthermore, pH changes or the release of exudates may facilitate the dissolution of some soil-P pools, potentially leading to an increase in available P in the entire rhizosphere, while a depletion occurs only in the direct vicinity of the roots (Hinsinger 2001; Kuppe et al. 2022). This finding indicates that P replenishment took place, overlaying the effect of P depletion through plant P uptake. Given the strong decrease in labile native soil P in the bulk soil, P replenishment may have occurred in the rhizosphere through the desorption of native soil P from the bulk soil.

In our experiment, the native soil P concentration in the resin-P fraction significantly increased under high-P conditions, whereas no change was detected under low-P conditions. This finding indicates that while the overall distribution of organic and inorganic labile P pools in the rhizosphere remained relatively unchanged upon plant growth, some shifts occurred when the origins of P according to fertilizer- or native soil-P were considered. Therefore, although no significant changes were detected in the separate inorganic or organic resin P pools, the overall balance indicated an increase in organic P in the resin-P fraction (Suppl Tables S2 and S4). Indeed, the enhanced uptake of native soil P upon P fertilization that was observed in Mundschenk et al. (2024) could be explained by the increase in native soil P or organic P in the most labile P fractions observed in this study. This suggests the solubilization or mineralization of native soil and organic P forms in the rhizosphere, which could function as a P source to replenish the soluble orthophosphate pool for the plant (Richardson 2001).

In the rhizosphere, we observed a strong depletion of organic NaOH-P, which indicates that substantial mineralization of moderately labile organic P took place. Concomitantly, an even more pronounced increase in inorganic NaOH-P was detected in the rhizosphere. In contrast, only a small but significant increase in the NaOH-P organic fraction was observed in the bulk soil, most likely because the diffusion of root-derived low-molecular-weight organic compounds into the bulk soil increased microbial activity (Schwalm et al. 2024). A decrease in organic NaOH-P was also found by Chen et al. (2002) in the rhizosphere of radiata pine and by Dissanayaka et al. (2015) in the rhizosphere of white lupine in an Andosol. The strong decline in organic NaOH-P in the rhizosphere might be explained by the release of phosphatases by plant roots or microbes, as well as by pH changes in the rhizosphere that facilitate P release from Al- or Fe-associated organo-minerals (Gahoonia et al. 1992; George et al. 2002a; Gérard 2016; Spohn 2020).

The release of phosphatases by plants or microorganisms leads to a mineralization of organic P, making it potentially available to plants (George et al. 2002b; Tian et al. 2021). George et al. (2006) associated the depletion of organic NaOH-P in two P-deficient Oxisols with increased extracellular acid phosphatase activity in the rhizosphere of thitonia and transgenic clover. Additionally, other authors reported the upregulation of phosphatase genes in rice in response to P deficiency (Pariasca-Tanaka et al. 2009; Gao et al. 2017). In contrast, Hedley et al. (1994) reported increased phosphatase activities near the root plane of rice but did not find a corresponding depletion of organic P. Overall, evidence that increased phosphatase secretion enables a greater PAE in various upland rice genotypes under P-limiting conditions is lacking (Rakotoson et al. 2020; Shimamura et al. 2021).

Changes in pH resulting from root exudation or root respiration are frequently discussed as potential factors contributing to increased P availability (George et al. 2002a; Kuppe et al. 2022; Liu et al. 2022). In another experiment of ours with similar experimental conditions, a small but consistent increase in pH of 0.3 was found in the rhizosphere compared to bulk soil (unpublished data). George et al. (2002a) linked a decrease in both, inorganic and organic NaOH-P concentrations in the rhizosphere of Tephrosia to rhizosphere alkalinization followed by decreased solubility of Fe and Al, which could have led to the release of precipitated or sorbed P. A positive effect on P availability during rhizosphere alkalization was also reported by Barrow (2017) and Kuppe et al. (2022). Further research is needed to investigate the impacts of plant phosphatase release, pH changes, and beneficial microbial colonization of the rhizosphere on the mobilization of moderately labile organic P.

In our experiment, the substantial mineralization of organic P in the NaOH-P fraction (i.e. depletion) was accompanied by an even greater increase in inorganic P in this fraction. Considering the extremely high P sorption of the tested Andosol (Mundschenk et al. 2024), it is likely that the mineralized organic P was rapidly sorbed to the soil matrix and recovered as moderately labile inorganic P. George et al. (2006) also proposed this mechanism, identifying a depletion of organic NaOH and a corresponding increase in inorganic NaOH-P. The authors associated this observation with the strong P sorption observed in the studied Oxisol.

We previously showed that DJ123 outperformed Nerica4 in terms of P acquisition (Mundschenk et al. 2024). Under high-P conditions, this was explained by the better access of DJ123 to both fertilizer and native soil-P, whereas under low-P conditions, DJ123 accessed more native soil-P than did Nerica4. Additionally, DJ123 was more efficient at acquiring native soil P per unit root mass under low- and high-P conditions (Mundschenk et al. 2024). These differences in P acquisition strategies were reflected in the shifts observed in this study within the soil P fractions, particularly under high-P conditions. In this study, genotypic differences between DJ123 and Nerica4 under low-P conditions were limited to a greater decrease in the bulk soil in inorganic H_2_SO_4_-P in Nerica4 than in DJ123. The lack of observed differences could be attributed to the short duration of the experiment and the extremely low P uptake by the plants, as indicated by the average plant P concentration of 0.06%.

Nerica4 significantly reduced the amount of easily exchangeable inorganic and fertilizer-derived P in the resin-P fraction in the rhizosphere, whereas no relative change was observed for DJ123. The strong decrease in the labile P pools (resin-P and NaHCO_3_-P) suggested that Nerica4 acquired easily available P and strongly depleted it in the rhizosphere but that P mobilization mechanisms were not sufficient to replenish the labile pool. Furthermore, the depletion of fertilizer-P in the resin-P fraction exceeded the depletion of inorganic P, indicating that both inorganic and organic P were depleted by Nerica4. Although the most common perspective is that plants can take up P only as inorganic orthophosphate ions (${\text{H}}_{2}{\text{PO}}_{4}^{-}$ or ${\text{H}}_{2}{\text{PO}}_{4}^{2-}$) from the soil solution (Hinsinger 2001), there is some evidence that plants can also absorb organic P forms such as nucleic acids (Sulieman and Mühling 2021). However, as mentioned earlier, the organic resin-P fraction is rarely measured by researchers, making broader comparisons with existing literature challenging.

In contrast to Nerica4, the rhizosphere of DJ123 showed a significant increase in organic and native soil P in the resin-P fraction. The maintenance of easily available inorganic P concentrations and the elevated concentrations of easily exchangeable organic or native soil P in the rhizosphere indicate that DJ123 possesses some traits that increase the solubilization or mineralization of P from more recalcitrant P pools. In addition to the previously mentioned pH changes and phosphatase secretion, other mechanisms, such as the exudation of organic anions, such as carboxylates, could contribute to organic P solubilization. Organic anions play a major role in breaking down soil organic matter by stimulating the disruption of metal bonds, thus releasing organic P forms (Clarholm et al. 2015). For example, Oburger et al. (2011) evaluated the efficiency of carboxylates in solubilizing phosphate and reported that it was greatest in soils with moderate to high levels of anionic binding sites, such as Al- and Fe-oxy(hydr)oxides. However, while this study focused on inorganic P solubilization, further research is needed to clarify the role of organic acid anions in the release of organic P in the rhizosphere of upland rice genotypes with contrasting PAEs. Assuming that crops primarily take up inorganic P, readily exchangeable organic P would first have to be mineralized. This mineralization would need to occur in the immediate root vicinity to ensure plant uptake while avoiding sorption by soil particles or microbial consumption (Zhang et al. 2024). Root-exuded enzymes, such as nucleases capable of hydrolyzing nucleic acids, can facilitate the breakdown of organic P into inorganic P, making it available for crop uptake (Zhang et al. 2024). Notably, our considerations assume that organic resin-P is truly of organic origin and not derived from condensed inorganic P. Therefore, further research is needed to characterize the extracted ‘organic’ resin-P pool in the rhizosphere of upland rice genotypes. Moreover, it is noteworthy that DJ123’s overall increased root growth compared to Nerica4 contributes to its superiority under P-limiting conditions (Schwalm et al. 2024). The main questions for future studies are therefore whether DJ123 can mobilize native soil and organic P forms in particular or enable habitat conditions for enhanced microbial activity and P mobilization and whether labile organic P forms can be directly taken up by P-efficient upland rice genotypes.

## Conclusions

We found that moderate P fertilization improved plant growth, facilitating processes that presumably increased the mobilization of native soil P. Our findings suggest substantial mineralization in the rhizosphere in the organic NaOH-P fraction, which was accompanied by an even greater relative increase in the inorganic NaOH-P fraction. This finding indicates that P mineralizing processes increased with increasing plant growth but that transformation into more plant-available P forms was likely constrained by high P sorption. Further research is needed to understand the factors driving increased mineralization of organic NaOH-P in the rhizosphere of upland rice genotypes and to determine whether mobilized P can become accessible to plants. This study was the first to evaluate changes in organic and native soil resin-P in the rhizosphere, revealing the significant contributions of these P forms to the total resin-P pool. These findings suggest that organic resin-P could serve as a readily available P source for crops. Understanding how, and to what extent, native soil or organic resin-P supports P nutrition in upland rice remains an important area for future research. Moreover, genotypic differences emerged under high-P conditions, with the more P efficient genotype DJ123 differing from Nerica4: instead of a depletion of the most readily available inorganic and fertilizer resin-P forms, an increase in the levels of native soil and organic resin-P took place for DJ123, potentially replenishing the available P pool. Further research is needed to understand the mechanisms behind these findings to help explain the enhanced P acquisition efficiency of DJ123 and offer insights for improving P acquisition in other crop genotypes.

## Data Availability

The datasets generated during the current study are available from the corresponding author on reasonable request.

## Abbreviations

PdfF: P derived from fertilizer
PdfS: P derived from native soil
Pi: Inorganic P
Po: Organic P
S.A.: Specific activity
T_0_: Initial soil one day after fertilization
T_1_: Soil at harvest after 34 days of plant growth
